# The Origin and Formation of the Dental Follicle

**Published:** 1879-11

**Authors:** 


### BIBLIOGRAPHICAL.

---

*The Origin and Formation of the Dental Follicle.*—The first memoir on the Development of the Teeth, by Drs. Charles Legros, and E. Magitot.

A translation from the French, with introduction and notes, by M. S. Dean, of Chicago. Authorized and reviewed by Dr. Magitot. Publishers: Jansen, McCleiry & Co., Chicago, 1880.

Dr. Dean, in presenting this admirable translation of a most interesting treatise on Dental Histology, has made a valuable addition to dental literature, which we have no doubt will be received as the labor in its compilation deserves, and the talent required merits. The need for such a treatise as Dr. Dean has presented, and the reputation of such distinguished histologists as Drs. Magitot and Legros, will ensure a welcome for this volume, which must reflect the highest credit on the translator as well as the authors.

The conversational style in which the work is written has the advantage of rendering what many might, perhaps, term a dry scientific subject, interesting as well as instructive, and may have a tendency to induce those who take but little interest in abstruse science to carefully peruse it, and thereby receive the benefit which such a work must confer upon all in any manner interested in the subject. To the dental student it will afford much instruction, and render easy of comprehension one of the most difficult subjects he is required to understand at the commencement of his Collegiate Course.

The work is finely illustrated and gotten up in a style, so far as type, paper and binding are concerned, which reflects credit upon the Publishers.

